# Isoform-Specific Dominant-Negative Effects Associated with hERG1 G628S Mutation in Long QT Syndrome

**DOI:** 10.1371/journal.pone.0042552

**Published:** 2012-08-02

**Authors:** Matthew R. Stump, Qiuming Gong, Zhengfeng Zhou

**Affiliations:** Division of Cardiovascular Medicine, Department of Medicine, Oregon Health & Science University, Portland, Oregon, United States of America; University of Milan, Italy

## Abstract

**Background:**

Mutations in the *human ether-a-go-go-related gene 1* (*hERG1*) cause type 2 long QT syndrome (LQT2). The *hERG1* gene encodes a K^+^ channel with properties similar to the rapidly activating delayed rectifying K^+^ current in the heart. Several hERG1 isoforms with unique structural and functional properties have been identified. To date, the pathogenic mechanisms of LQT2 mutations have been predominantly described in the context of the hERG1a isoform. In the present study, we investigated the functional consequences of the LQT2 mutation G628S in the hERG1b and hERG1a_USO_ isoforms.

**Methods:**

A double-stable, mammalian expression system was developed to characterize isoform-specific dominant-negative effects of G628S-containing channels when co-expressed at equivalent levels with wild-type hERG1a. Western blot and co-immunoprecipitation studies were performed to study the trafficking and co-assembly of wild-type and mutant hERG1 isoforms. Patch-clamp electrophysiology was performed to characterize hERG1 channel function and the isoform-specific dominant-negative effects associated with the G628S mutation.

**Conclusions:**

The non-functional hERG1a-G628S and hERG1b-G628S channels co-assembled with wild-type hERG1a and dominantly suppressed hERG1 current. In contrast, G628S-induced dominant-negative effects were absent in the context of the hERG1a_USO_ isoform. hERG1a_USO_-G628S channels did not appreciably associate with hERG1a and did not significantly suppress hERG1 current when co-expressed at equivalent ratios or at ratios that approximate those found in cardiac tissue. These results suggest that the dominant-negative effects of LQT2 mutations may primarily occur in the context of the hERG1a and hERG1b isoforms.

## Introduction

Long QT syndrome (LQTS) is a cardiac disorder characterized by QT prolongation and an increased risk of severe ventricular arrhythmias that can result in sudden death [1], [2]. Mutations in the *human ether-go-go-related gene 1* (*hERG1*) result in LQTS type 2 (LQT2) and account for 25 to 40% of genotyped cases of LQTS [3], [4], [5]. To date, over 500 LQT2 mutations have been identified. The *hERG1* gene encodes the pore-forming subunit of the rapidly activating delayed rectifier K^+^ channel (*I_Kr_*) in the heart, which contributes to the repolarization of the cardiac action potential [6], [7]. Most LQT2 mutations are loss of function mutations that result in decreased hERG1 current levels and several mutations are known to dominantly suppress wild-type hERG1 current [8], [9].

Several hERG1 isoforms have been identified in the heart and are known to modulate *I_Kr_*. The first cloned hERG1 isoform, hERG1a, contains 1159 amino acids and represents the full-length hERG1 channel protein [10]. hERG1a channels exhibit voltage-dependent activation and undergo inactivation at positive depolarizing potentials [6], [7], [11]. The N-terminus of hERG1a plays an important role in channel deactivation, while several regions in the C-terminus contribute to channel assembly and trafficking. The *hERG1* gene has an alternate transcription start site within intron 5 that generates a transcript encoding the hERG1b isoform [12], [13], [14]. The 376 N-terminal residues of hERG1a channels are replaced by 36 unique residues in hERG1b resulting in channels that exhibit accelerated rates of channel deactivation. Recent studies have shown that hERG1b co-assembles with hERG1a and it was suggested that the heteromeric channels underlie native ventricular *I_Kr_* [15]. A third hERG1 isoform, hERG1a_USO_, arises from the inefficient splicing of intron 9 resulting in premature termination and polyadenylation within intron 9 [16]. The 359 C-terminal residues of hERG1a are replaced by 88 unique residues in hERG1a_USO_ channels. In contrast to hERG1a and hERG1b, hERG1a_USO_ is trafficking defective and does not generate hERG1 current when expressed in mammalian cells [17], [18]. hERG1a_USO_ has been shown to co-assemble with hERG1a, interfering with the trafficking of the functional isoform, when over-expressed relative to hERG1a in heterologous systems [18].

The majority of LQT2 mutations occur within coding regions common to hERG1a, hERG1b, and hERG1a_USO_, however, the functional consequences of these mutations, including G628S, have been primarily characterized in hERG1a. hERG1a-G628S channels have been found to be non-functional and to dominantly suppress wild-type hERG1 current [8]. In the present study we characterized the functional properties of the G628S mutation in the context of hERG1b and hERG1a_USO_ isoforms. We developed a double-stable, mammalian expression system using a recombinase-mediated recombination strategy to study the isoform-specific dominant-negative effects of the G628S mutation. We found that the assembly of hERG1a with hERG1a-G628S or hERG1b-G628S resulted in the dominant suppression of hERG1 current. In contrast, hERG1a_USO_-G628S did not readily associate with hERG1a, and consequently, did not suppress hERG1 current when co-expressed at a 1∶1 ratio or at ratios that approximate those found in cardiac tissue. These results suggest that pathogenic consequences of LQT2 mutations may primarily occur in hERG1a and hERG1b.

## Results

### Design of the Flp-Cre expression system

**Figure 1:**
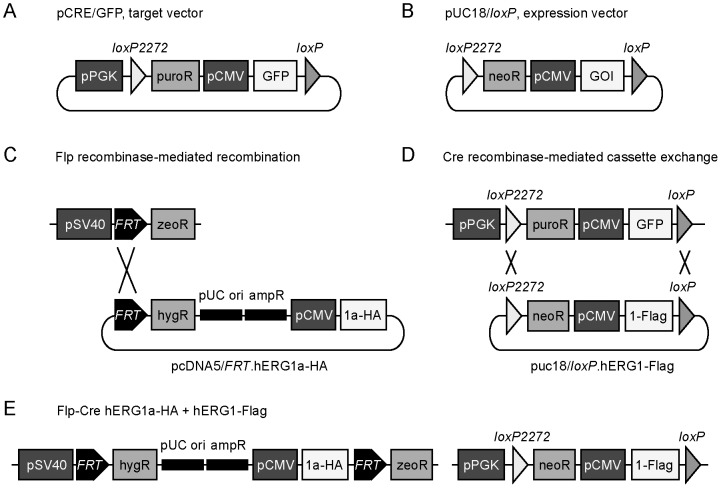
Schematic illustrating the generation of the double-stable Flp-Cre cell line. (A) The pCRE/GFP target vector was designed to introduce the *loxP2272/loxP* recombination cassette into Flp-In-293 cells. The recombination cassette is comprised of incompatible *loxP2272* and *loxP* sites flanking the puromycin resistance gene (puroR) and green fluorescent protein (GFP). The cytomegalovirus promoter (pCMV) drives the expression of GFP. The phosphoglycerate kinase promoter (pPGK) was inserted upstream of the *loxP2272* site to control the expression of the puroR. (B) The pUC18/*loxP* expression vector was designed to stably integrate the gene of interest (GOI) at the *loxP2272/loxP* cassette of Flp-Cre cells using a Cre recombinase-mediated cassette exchange approach. The *loxP2272*/*loxP* cassette of the expression vector contains the neomycin resistance gene (neoR) and the CMV promoter which controls the expression of the GOI. The promoter for neoR expression, pPGK, is only provided following recombination at the *loxP2272/loxP* site within Flp-Cre cells. (C) Flp recombinase-mediated recombination is used to introduce the gene of interest at the *FRT* target site. The *FRT* target site is comprised of the zeocin resistance gene (zeoR) under the control of the simian virus 40 early promoter (pSV40). HA-tagged hERG1a (1a-HA) was stably introduced by co-transfection with the pcDNA5/*FRT* expression vector and a Flp recombinase expression vector according to the Flp-In System protocol (Invitrogen). Additional components of the pcDNA5/*FRT* expression vector include the hygromycin resistance gene (hygR), pUC ori, and the ampicillin resistance gene (ampR). (D) Cre recombinase-mediated cassette exchange is used to introduce the gene of interest at the *loxP2272/loxP* site. Flag-tagged hERG1 (1-Flag) constructs were stably integrated into the Flp-Cre hERG1-HA stable cell line by co-transfection of the pUC18/*loxP* expression vector and a Cre recombinase expression vector. (E) The double-stable Flp-Cre cell line contains a single copy of the HA-tagged hERG1a gene and the Flag-tagged hERG1 gene.

To characterize the functional properties and the dominant-negative effects of the G628S mutation in the hERG1a, hERG1b and hERG1a_USO_ isoforms, we developed a novel, double-stable, mammalian expression system (described in detail in the Materials and Methods, Fig. 1). Briefly, we stably introduced a *loxP2272/loxP* recombination cassette into Flp-In-293 cells using the pCRE/GFP target vector. To confirm that the *loxP2272/loxP* cassette had integrated at a single genomic locus we performed quantitative real-time PCR using primers specific to the zeocin gene, a component of the *FRT* cassette and to GFP, a component of the *loxP2272/loxP* cassette. The averaged GFP/zeocin ratio was 1.02±0.04 (n = 3). Since Flp-In-293 cells contain a single copy of the *FRT* site, the real-time PCR results indicate that Flp-Cre cells contain a single copy of the *loxP2272/loxP* site.

**Figure 2:**
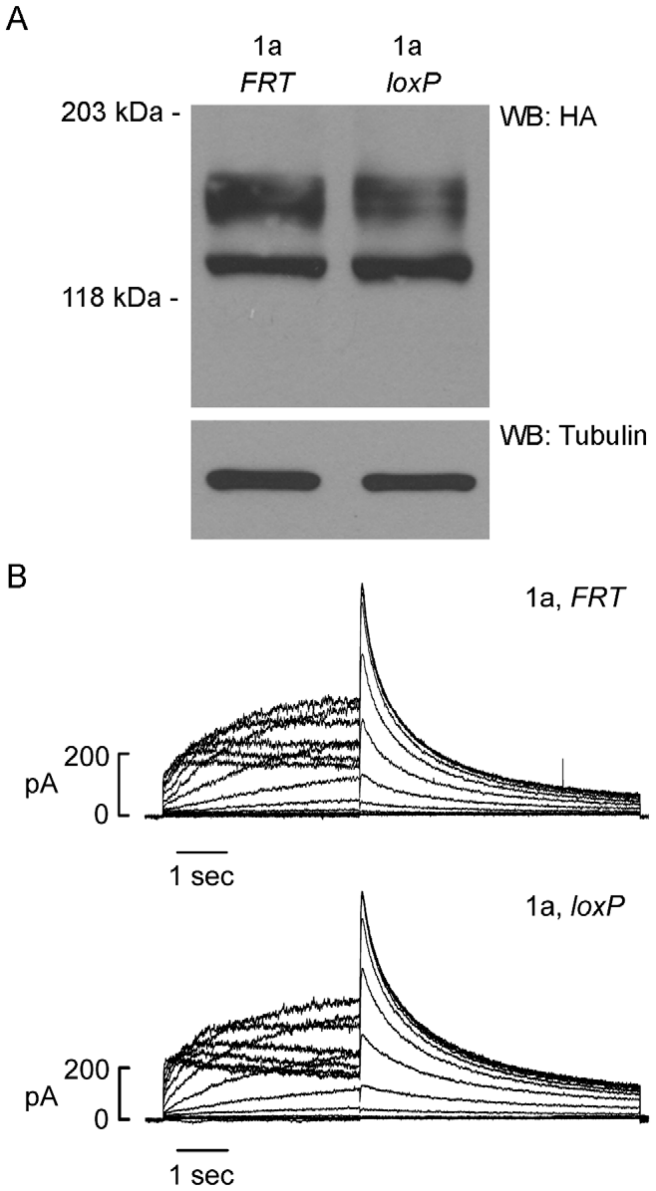
Biochemical and functional analysis of hERG1a expressed from the *FRT* and *loxP2272/loxP* sites in Flp-Cre cells. (A) Flp-Cre cells stably expressing HA-tagged hERG1a (1a) from the *FRT* or the *loxP2272/loxP* sites were detected by western blot with the anti-HA antibody. Tubulin was used as a loading control. Results shown are representative of three independent experiments. (B) Representative current traces recorded from cells stably expressing hERG1a from the *FRT* and the *loxP2272/loxP* sites. The channels were activated by depolarizing steps between −70 and 60 mV from a holding potential of −80 mV and tail current was recorded upon repolarization to −50 mV.

We performed western blot and patch-clamp analyses to compare the expression of HA-tagged hERG1a channels stably integrated at the *FRT* or the *loxP2272/loxP* site (Fig. 2). hERG1a channel proteins integrated at both sites were expressed as the core-glycosylated, immature form (135 kDa) and the fully-glycosylated, mature form (155 kDa) (Fig. 2A). Quantitative densitometry revealed that the ratio of hERG1a expressed from the *loxP2272/loxP* relative to the *FRT* site was 1.11±0.18. Functional studies revealed similar levels of hERG1a current recorded from the two cell lines (Fig. 2B). The averaged peak tail current amplitudes of hERG1a expressed from the *FRT* and *loxP2272/loxP* sites were 21.3±1.9 pA/pF (n = 8) and 23.6±2.6 (n = 10, *P*>0.05), respectively. These results indicated that the Flp-Cre system would permit the characterization of LQT2-induced dominant-negative effects at a 1∶1 expression ratio when co-transfected with wild-type and mutant hERG1 channel constructs.

### Functional properties of hERG1 isoforms containing the LQT2 mutation G628S

**Figure 3:**
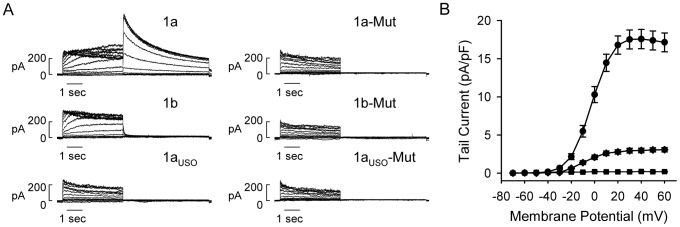
Functional analysis of wild-type and mutant hERG1 isoforms. (A) Representative currents recorded from Flp-Cre cells stably expressing Flag-tagged wild-type or G628S-mutant (Mut) hERG1a (1a), hERG1b (1b), and hERG1a_USO_ (1a_USO_) from the *loxP2272/loxP* site. (B) Current-voltage plot of the peak tail current amplitude measured at −50 mV following depolarizing voltages from −70 to 60 mV recorded from 1a (circles, n = 21), 1b (diamonds, n = 6) and 1a_USO_ (squares, n = 6) channels. The voltage-clamp protocol is given in the legend of Fig. 2.

To characterize the functional properties of the G628S mutation we generated stable Flp-Cre cell lines expressing Flag-tagged wild-type or mutant hERG1 isoforms from the *loxP2272/loxP* site. Representative current traces of wild-type and mutant hERG1 isoforms are shown in Fig. 3A. hERG1a and hERG1b channels exhibited voltage-dependent activation and inward rectification at more positive depolarizing potentials. hERG1 current was not detected in cells transfected with hERG1a_USO_ or with mutant hERG1a, hERG1b or hERG1a_USO_ isoforms. The current-voltage plot shown in Fig. 3B compares the average peak tail current density of the wild-type hERG1 isoforms. The maximum tail densities of hERG1a and hERG1b channels were 17.5±1.2 pA/pF (n = 21) and 3.0±0.3 pA/pF (n = 6). The decrease in the tail current amplitude of hERG1b is consistent with previous reports and is caused by rapid channel deactivation and decreased trafficking of the hERG1b isoform to the cell-surface [19], [20].

**Figure 4:**
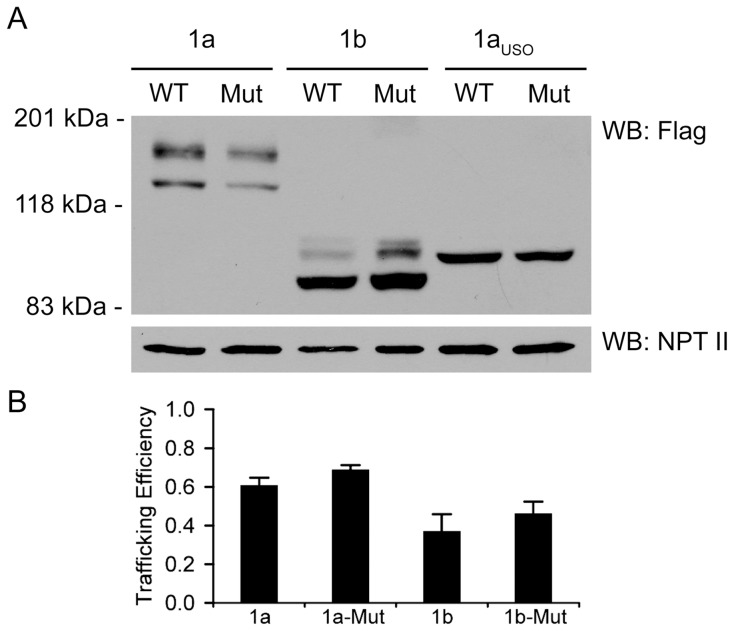
Western blot analysis of wild-type and mutant hERG1 isoforms. (A) Proteins from Flp-Cre cells stably transfected with Flag-tagged wild-type (WT) or G628S-mutant (Mut) hERG1a (1a), hERG1b (1b) and hERG1a_USO_ (1a_USO_) isoforms from the *loxP2272/loxP* site were detected with the anti-Flag antibody. Mature, fully-glycosylated hERG1a and hERG1b channels are 155 kDa and 105 kDa, respectively; immature, core-glycosylated hERG1a, hERG1b, and hERG1a_USO_ channels are 135 kDa, 90 kDa, and 100 kDa, respectively. Neomycin phosphotransferase II (NPT II) was detected by a polyclonal anti-NPT II antibody. (B) The trafficking efficiency is plotted as the percentage of the normalized upper, mature band to the total normalized hERG1 protein. The data are plotted as mean ± SEM. Results shown are representative of three independent experiments.

The trafficking properties of hERG1 isoforms were determined by western blot analysis using anti-Flag antibody (Fig. 4A). Wild-type and mutant hERG1a channels were expressed as the immature and mature channel proteins as described in Fig. 2A. Wild-type and mutant hERG1b channels were expressed as the immature (90 kDa) and the mature (105 kDa) channel proteins. As has been previously shown, hERG1b protein was primarily expressed as the immature band indicating that hERG1b does not traffic as efficiently as hERG1a channels. The low trafficking efficiency of hERG1b channel has been attributed to the presence of an endoplasmic reticulum retention signal found in the unique N-terminus of the isoform [20]. The G628S mutation does not appear to alter the trafficking properties of the wild-type hERG1a and hERG1b channels. The trafficking efficiency of wild-type and mutant hERG1a and hERG1b was defined as the ratio of the upper band to the total hERG1 protein. As shown in Fig. 4B, the G628S mutation does not significantly alter the trafficking efficiency of hERG1a or hERG1b (n = 3, *P*>0.05). Wild-type and mutant hERG1a_USO_ were expressed as the immature channel protein at 100 kDa indicating that the isoform is trafficking deficient [18].

### Isoform-specific dominant-negative effects

**Figure 5:**
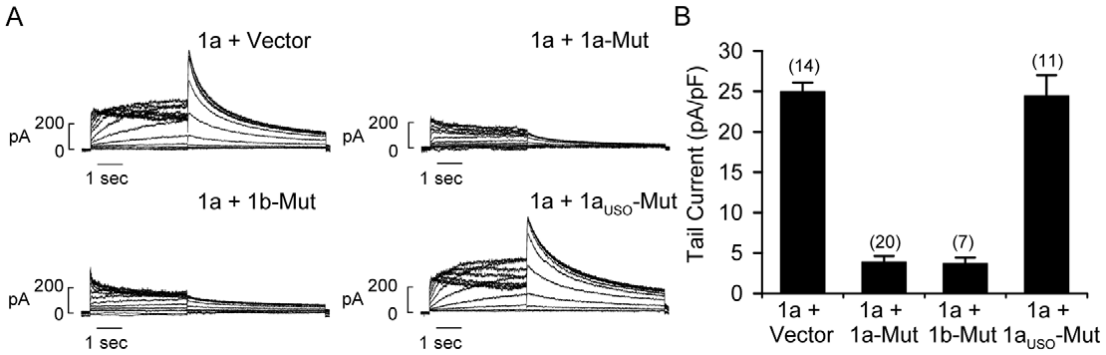
Isoform-specific dominant-negative effects. (A) Representative current traces from Flp-Cre cells stably co-expressing hERG1a-HA (1a) with G628S-mutant hERG1a-Flag (1a-Mut), hERG1b-Flag (1b-Mut), or hERG1a_USO_-Flag (1a_USO_-Mut). Flp-Cre cells co-transfected hERG1a and empty vector were used as a control. hERG1 current was recorded using the protocol described in the Fig. 2 legend. (B) Averaged hERG1 tail current amplitude measured at −50 mV following depolarizing voltages to 30 mV. The number of cells is shown in parentheses.

To characterize the dominant-negative effects of the G628S mutation we generated cell lines co-expressing wild-type hERG1a and mutant hERG1 isoforms at a 1∶1 ratio. Double-stable Flp-Cre cell lines were generated with hERG1a-HA at the *FRT* site and empty vector, hERG1a-G628S-Flag, hERG1b-G628S-Flag, or hERG1a_USO_-G628S-Flag at the *loxP2272/loxP* site. The dominant-negative suppression of hERG1 current by the mutant hERG1a and hERGb channels is clearly shown in Fig. 5. The average peak tail current density recorded from cells co-transfected with hERG1a + vector was 25.0±1.1 pA/pF (n = 14). When hERG1a was co-expressed with hERG1a-G628S or hERG1b-G628S we observed a significant decrease in hERG1 current as compared to hERG1a + vector control (3.9±0.7 pA/pF, n = 20 and 3.7±0.7 pA/pF, n = 7, respectively, *P*<0.05). In contrast, current recorded from cells expressing hERG1a + hERG1a_USO_-G628S cells was not significantly different from the control (24.5±2.5 pA/pF, n = 11, *P*>0.05). These results strongly suggest that mutant hERG1a and hERG1b may be primarily responsible for the pathological consequences of LQT2 mutations.

### Isoform-specific assembly of hERG1 isoforms

**Figure 6:**
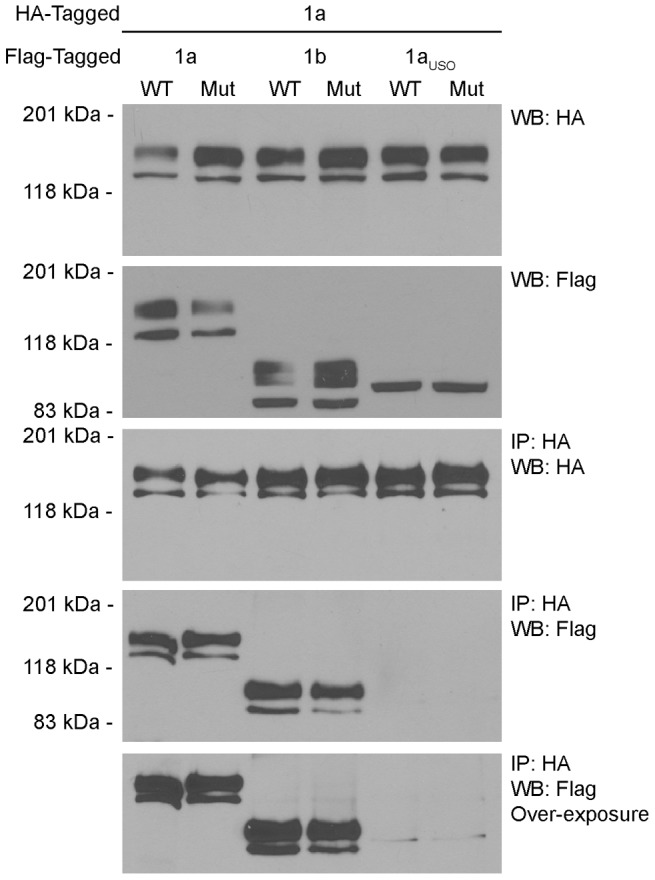
Co-assembly of hERG1 isoforms. Proteins from Flp-Cre cells co-expressing HA-tagged hERG1a (1a) with Flag-tagged wild-type (WT) or mutant (Mut) 1a, hERG1b (1b) or hERG1a_USO_ (1a_USO_) channels were detected by western blot with the anti-HA and the anti-Flag antibody (upper two panels). Co-assembly of hERG1 isoforms was determined by immunoprecipitation with the anti-HA antibody and detection with the anti-Flag and the anti-HA antibody (lower three panels). Results shown are representative of three independent experiments.

To determine the underlying mechanism of the isoform-specific dominant-negative effects of the G628S mutation we determined whether hERG1a co-assembled with the wild-type and mutant hERG1 isoforms (Fig. 6). The expression of the hERG1 channels from the double-stable cell lines used in Fig. 5 was confirmed by western blot analysis. Co-assembly of the differentially-tagged hERG1 isoforms was determined by immunoprecipitating hERG1a-HA with the anti-HA antibody and detecting Flag-tagged hERG1 channels by western blot with the anti-Flag antibody. Western blot analysis with the anti-HA antibody revealed that hERG1a was efficiently immunoprecipitated. As expected, hERG1a-G628S and hERG1b-G628S readily associated with both mature and immature hERG1a channels. The co-assembly of the G628S-containing 1a and 1b isoforms with wild-type hERG1a gives rise to the dominant-negative suppression of hERG1 current. In contrast, wild-type and mutant hERG1a_USO_ proteins showed minimal association with hERG1a as evidenced by the presence of faint bands corresponding to the hERG1a_USO_ channel that were observed only upon over-exposure. To test whether the association between hERG1a and hERG1a_USO_ was destabilized by the detergent used in the immunoprecipitation buffer we repeated the immunoprecipitation experiments using buffer in which the Triton X-100 detergent was replaced with the NP40 detergent (Fig. S1). Similar faint bands were observed upon over-exposure of the blot. These results support the conclusion that the association between hERG1a and hERG1a_USO_ is significantly weaker than the association of 1a/1a and 1a/1b isoforms.

To further characterize trafficking of the mutant hERG1a_USO_ channels we performed immunofluorescence studies using Flp-Cre cells stably expressing HA-tagged hERG1a and Flag-tagged hERG1a-G628S or Flag-tagged hERG1a_USO_-G628S channels (Fig. S2). Wild-type and mutant hERG1a exhibited a diffuse staining pattern visible throughout the cell including the cell processes. In contrast, hERG1a_USO_-G628S was not observed in the cell processes, an indicative of trafficking deficiency. These results are consistent with the western blot, patch clamp and co-immunoprecipitation studies and support the conclusion that hERG1a_USO_-G628S is trafficking defective and does not readily associate with hERG1a.

**Figure 7:**
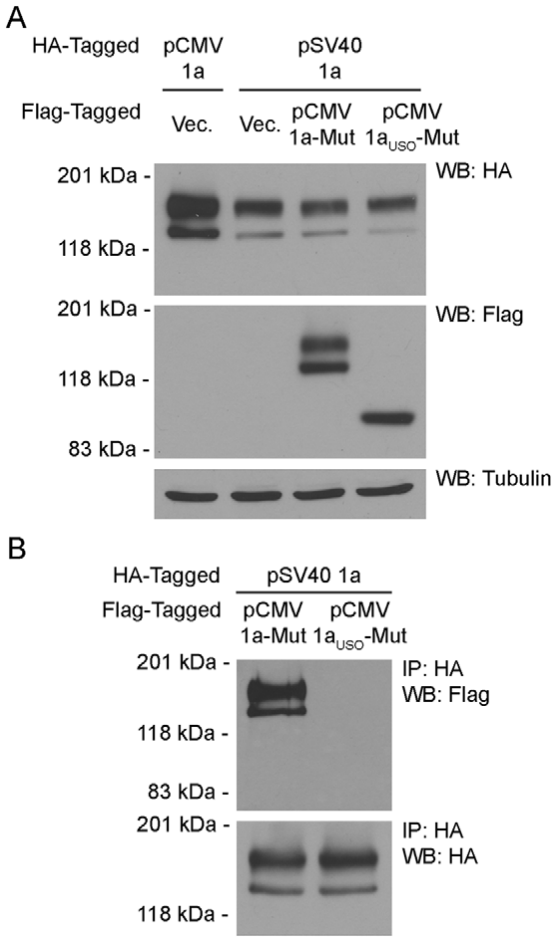
Co-assembly of hERG1 isoforms expressed at ratios approximating those found in the heart. (A) Proteins from Flp-Cre cells expressing HA-tagged hERG1a (1a) from the weak SV40 promoter (pSV40) at the *FRT* site and Flag-tagged hERG1a-G628S (1a-Mut) or hERG1a_USO_-G628S (1a_USO_-Mut) from the CMV promoter (pCMV) at the *loxP2272/loxP* site were detected by western blot with the anti-HA (upper panel) and the anti-Flag antibody (middle panel). Flp-Cre cells expressing HA-tagged hERG1a from the CMV or SV40 promoter at the *FRT* site and empty vector in *loxP2272/loxP* site were used as controls; tubulin was used as a loading control (lower panel). (B) Co-assembly of hERG1 isoforms was determined by immunoprecipitation using the anti-HA antibody followed by western blot with the anti-Flag and the anti-HA antibody. Results shown are representative of three independent experiments.

Previous quantitative real-time PCR and RNase protection assay studies have shown that hERG1a_USO_ mRNA is present at approximately twice the level of hERG1a in the heart [16], [17]. We wondered whether hERG1a_USO_ exerted a dominant-negative effect on hERG1 current when co-expressed with wild-type hERG1a at ratios that approximate those found in the heart. To modify the relative expression of hERG1 isoform in the Flp-Cre cell line we replaced the strong CMV promoter of the pcDNA5/*FRT* expression vector with the weaker SV40 promoter, effectively decreasing the level of the hERG1a-HA expressed from the *FRT* site following the stable transfection of Flp-Cre cells. We generated Flp-Cre cell lines expressing hERG1a-HA from the SV40 promoter at the *FRT* site and empty vector, hERG1a-G628S-Flag or hERG1a_USO_-628-Flag from the CMV promoter at the *loxP2272/loxP* site. The Flp-Cre cell line containing hERG1a-HA expressed from the CMV promoter at the *FRT* site and empty vector at the *loxP2272/loxP* site (described in Fig. 5) was used a control. Wild-type hERG1a channels were detected by western blot using the anti-HA antibody (Fig. 7A, top panel). The relative amounts of hERG1a channel proteins expressed from the two promoters are clearly shown. The hERG1a expressed from the SV40 promoter was found to be 39±7% of that expressed from the CMV promoter. Re-probing the membrane with the anti-Flag antibody revealed the presence of hERG1a-G628S and hERG1a_USO_-G628S channel proteins expressed from the CMV promoter (Fig. 7A middle panel).To characterize the physical association between the wild-type channel and the mutant isoforms we immunoprecipitated hERG1a-HA with the anti-HA antibody and performed western blot analysis with the anti-Flag and the anti-HA antibody (Fig. 7B). As expected, hERG1a-G628S was found to readily associate with wild-type hERG1a channels. In contrast, hERG1a_USO_-G628S did not co-assemble with hERG1a at levels approximating cardiac hERG1a:hERG1a_USO_ ratios.

**Figure 8:**
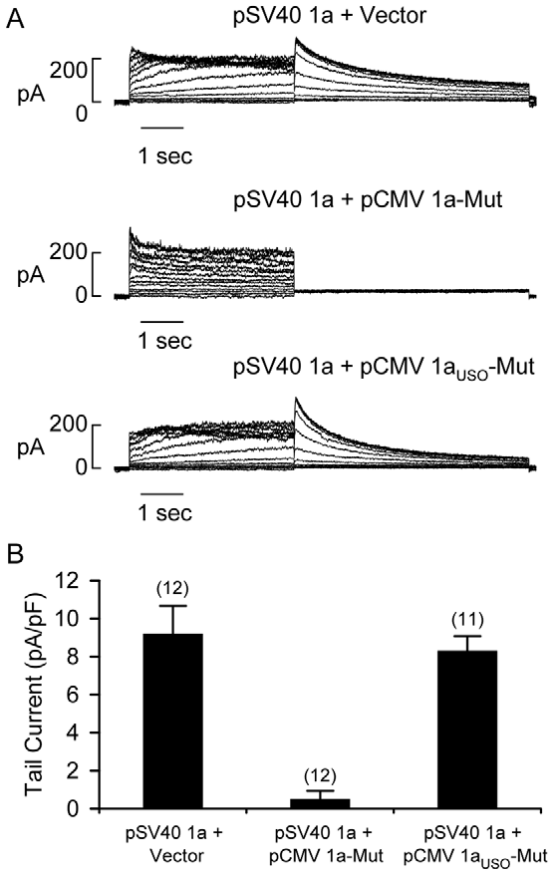
Functional analysis of hERG1a co-expressed with hERG1a_USO_-G628S at ratios approximating those found in the heart. (A) Representative current traces recorded from Flp-Cre cells expressing hERG1a (1a) from the weak SV40 promoter (pSV40) at the *FRT* site and empty vector, hERG1a-G628S (1a-Mut), or hERG1a_USO_-G628S (1a_USO_-Mut) from the CMV promoter (pCMV) at the *loxP2272/loxP* site. The patch-clamp protocol is given in the legend of Fig. 2. (B) Averaged tail current amplitude measured at −50 mV following depolarizing voltages from 30 mV. The number of cells is shown in parentheses.

We performed patch-clamp analysis to test whether hERG1 current was dominantly suppressed by the increased relative expression of hERG1a_USO_-G628S (Fig. 8). Flp-Cre cell lines co-expressing hERG1a from the SV40 promoter and empty vector or hERG1a-G628S from the CMV promoter were used as controls. Representative current trances are shown in Fig. 8A. The peak tail current density from cells expressing hERG1a + vector was 9.2±1.5 pA/pF (n = 12), approximately 63% less than the current generated from Flp-Cre cells expressing hERG1a from the strong CMV promoter (Fig. 5). The peak tail current recorded from cells expressing hERG1a + hERG1a-G628S was 0.5±0.4 pA/pF (n = 12, *P*<0.05 compared to hERG1a/vector control) indicating the near complete loss of hERG1 current (Fig. 8B). The co-expression of hERG1a and hERG1a_USO_, however, did not significantly alter hERG1 current levels compared to the control (8.3±0.8 pA/pF, n = 11, *P*>0.05) (Fig. 8B). These results suggest that mutant hERG1a_USO_ channels may not have a significant role in the pathogenesis of LQT2.

## Discussion

In this study we analyzed the isoform-specific effects of the G628S LQT2 mutation on the function, trafficking, assembly and dominant-negative properties of the hERG1a, hERG1b and hERG1a_USO_ isoforms. While the mutation did not alter the trafficking properties of hERG1a and hERG1b, it completely disrupted the function of the both isoforms. Wild-type and mutant hERG1a_USO_ channels were trafficking deficient and non-functional. The G628S-induced dominant-negative effects were observed in the context of hERG1a and hERG1b but not hERG1a_USO_ when co-expressed with wild-type hERG1a at equivalent ratios or at ratios that approximate those found in the heart. The Flp-Cre system allowed the equivalent stable expression of two hERG1 isoforms. This system represents an advantage over the co-expression of two genes from a single promoter using IRES-containing bicistronic vectors which often exhibit decreased expression of the second, IRES-dependent gene [21], [22].

The G628S mutation was one of the earliest loss-of-function LQT2 mutations to be characterized and shown to dominantly suppress the function of hERG1a when expressed in *Xenopus* oocytes [8]. Because of the mutation's severe pathogenic phenotype, it has since been widely used in the characterization of the function and dysfunction of hERG1a channels and *I_Kr_* [23], [24], [25]. Our results clearly show that hERG1b-G628S channels are dysfunctional and confer significant dominant-negative effects, comparable to those observed in hERG1a-G628S. hERG1a-G628S and hERG1b-G628S were both shown to decrease hERG1 current by 85% when co-expressed with hERG1a at a 1∶1 ratio. hERG1a and hERG1b differ structurally in the length and the composition of the N-terminus with hERG1b lacking several regions that contribute to the regulation of channel deactivation. These regions include the Per, Arnt, and Sim (PAS) domain that orients the N-terminus into close proximity to the S4–S5 linker and the cyclic nucleotide binding domain [26], [27], [28]. The absence of these N-terminal regulatory domains resulted in the accelerated deactivation rates observed in Fig. 3. While the relative expression level of hERG1b mRNA comprises less than 20% of the total hERG1 mRNA in the heart, studies in *Xenopus* oocytes have shown that these levels are sufficient to modulate hERG1 deactivation rates [19]. The dominant-negative effects of hERG1b-G628S shown in the present study underscore the pathological significance of LQT2 mutations in the hERG1b isoform.

The hERG1a_USO_-G628S isoform, however, did not dominantly suppress hERG1a current. The isoform-specific effects of the G628S mutation are likely due to the structural and functional properties of the different isoforms. The structural difference between hERG1a_USO_ and hERG1a or hERG1b is the length and the composition of the C-terminus. hERG1a_USO_ lacks the 359 C-terminal residues of hERG1a/1b which are replaced by 88 unique, presumably unstructured residues. This is due to alternative polyadenylation within intron 9 [16]. The C-terminal region of hERG1a/1b contains several domains that are important in hERG channel trafficking, assembly and function. The cyclic nucleotide binding domain of hERG1 plays an important role in the trafficking of hERG1 channels to the cell surface as LQT2 mutations occurring within this domain are known to disrupt channel trafficking [29], [30], [31], [32]. The truncation of the C-terminus deletes two additional C-terminal domains that contribute to hERG1a assembly. The region from residues 1018 to 1122 was reported to be necessary for the recapitulation of hERG1 current in C-terminally truncated channels [17], and a small, “tetramerizing coiled coil” domain (residues 1037 to 1074) was also proposed to mediate channel assembly [33]. The absence of all three of these domains likely underlies the dysfunction, defective trafficking, and impaired assembly of hERG1a_USO_ in the present studies. The absence of the full-length C-terminus in the hERG1a_USO_-G628S channels prevents the trafficking of the mutant channel and limits the association between the mutant channel and the full-length hERG1a channel, precluding the dominant-negative effects.

In our co-immunoprecipitation experiments, a small fraction of hERG1a_USO_ was found to associate with hERG1a (Fig. 6 and Fig. S1) indicating that the absence of the hERG1a C-terminus does not completely preclude channel assembly. The heteromeric assembly of hERG1a and hERG1a_USO_ under conditions in which the trafficking defective isoform is strongly over-expressed has been shown to regulate the surface expression of hERG1a [18]. A similar effect has been observed in voltage-gated Shaker B channels that lack the T1 tetramerization domain wherein removing T1 severely impairs assembly efficiency and increasing subunit concentration can partially overcome this assembly defect [34]. It is likely that the co-assembly of hERG1a and hERG1a_USO_ is partially driven by the over-expression of the hERG1a_USO_ subunit. The co-expression of differentially tagged hERG1 isoforms using the Flp-Cre system allowed us to directly compare the assembly of hERG1a and hERG1a_USO_ to homomeric hERG1a and heteromeric hERG1a and hERGb channels. The present results suggest that the assembly deficient hERG1a_USO_ isoform exerts minimal effects on the trafficking and function of hERG1a when co-expressed at ratios that approximate those found in cardiac tissue. An alternative mechanism of hERG1a current regulation by the mutually exclusive generation of either hERG1a or hERG1a_USO_ from the alternative processing of hERG1a pre-mRNA has recently been described [16].

In summary, our study highlights the differential disease-causing effects of LQT2 mutations in the three major hERG1 isoforms. The absence of hERG1a_USO_ dominant-negative effects is presumed to be due to trafficking deficiencies and the minimal co-assembly of between the hERG1a_USO_ and hERG1a. Although hERG1a_USO_ represents the hERG1 isoform with the highest expression level in the heart it appears that congenital LQT2 may primarily be caused by mutant hERG1a and hERG1b channels. The Flp-Cre expression system, developed in these studies, may prove to be useful in future studies of hERG1 channel function and dysfunction. Furthermore, the stable expression of two genes at an equivalent molar ratio using the Flp-Cre system may have broad applications in the characterization of protein function and dysfunction as well as in the analyses of protein-protein interactions.

## Materials and Methods

### hERG isoform cDNA constructs

hERG1b was cloned from the human heart Marathon-Ready cDNA (Clontech, Mountain View, CA) by PCR using 5′-GGG GAT CCG GCA GGC TGC AGG GAG CCA A-3′ (hERG1b forward) and 5′-GCC GAC ACG TTG CCG AAG ATG CTA-3′ (hERG1b reverse) primers. The PCR fragment was sequenced and subcloned into the backbone of hERG1a at BamHI and BglII sites to obtain the full-length hERG1b cDNA. The generation of hERG1a_USO_ cDNA has been described [16]. The generation of C-terminally Flag- and hemagglutinin (HA)-tagged hERG1 cDNA constructs have been described [32], [35]. The G628S mutation was generated by the Gene-Editor mutagenesis system (Promega, Madison, WI) and confirmed by sequencing.

### Design of the double-stable Flp-Cre cell line

To develop a double-stable mammalian expression system, we designed a target vector to introduce a *loxP2272/loxP* recombination cassette into the mammalian cell line Flp-In-293 (Invitrogen, Carlsbad, CA). Flp-In-293 cells are derived from the HEK-293 cell line by the integration of the Flp recombination target (*FRT*) site at a single genomic locus. The pCRE/GFP target vector was derived from pUC18 (Fermentas, Glen Burnie, MD) and contains a *loxP2272/loxP* recombination cassette comprised of incompatible *loxP2272* and *loxP* sites flanking the puromycin resistance gene and green fluorescence protein (GFP) (Fig. 1A). The 8 nt spacer region of the *loxP2272* and *loxP* sites differ by 2 nt precluding cross-recombination reactions [36]. Expression of the puromycin resistance gene is controlled by the PGK promoter inserted directly upstream of the *loxP2272* site, outside of the *loxP2272/loxP* cassette. The expression of GFP is controlled by the CMV promoter. Flp-In-293 cells were transfected with the pCRE/GFP vector (1.0 µg) using the Effectene method (Qiagen, Valencia, CA). Forty-eight hours post-transfection, the cells were cultured in DMEM and 10% FBS and 1 µg/ml puromycin. After fifteen days, single colonies stably expressing GFP were selected with cloning cylinders. Quantitative real-time PCR was performed to confirm the integration of a single copy of the *loxP2272/loxP* cassette. The resultant stable host cell line, Flp-Cre, contains a single copy of the *FRT* site and the *loxP2272/loxP* sites allowing Flp recombinase-mediated recombination and Cre recombinase-mediated cassette exchange reactions, respectively. Flp-Cre cells were cultured in DMEM and 10% fetal bovine serum and 1 µg/ml puromycin.

The pUC18/*loxP* expression vector (Fig. 1B) was designed to stably integrate a gene of interest at the *loxP2272/loxP* site of Flp-Cre cells. The vector contains *loxP2272* and *loxP* sites flanking the neomycin resistance gene and the gene of interest. The expression of the gene of interest was controlled by the CMV promoter. The promoter controlling the expression of the neomycin resistance gene is provided only upon recombination within the *loxP2272/loxP* cassette in the Flp-Cre cells. Stable integration of the gene of interest was achieved by co-transfecting Flp-Cre cells with the pUC18/*loxP* vector (0.1 µg) containing the gene of interest and a Cre recombinase expression vector (pBS185, 0.9 µg) [37] using the Effectene method. Forty-eight hours post-transfection, the cells were cultured in DMEM and 10% FBS and 400 µg/ml G418.

To generate double-stable cell lines, HA-tagged hERG1a was stably integrated at the *FRT* site of Flp-Cre cells by co-transfection with the pcDNA5/*FRT* expression vector containing hERG1a-HA (0.1 µg) and the Flp recombinase expression vector pOG44 (0.9 µg) using the Effectene method and selected with 100 µg/ml hygromycin (Fig. 1C). Flag-tagged wild-type and mutant hERG1 isoforms were then stably integrated at the *loxP2272/loxP* site of cells stably expressing hERG1a-HA using the Cre recombinase-mediated recombination approach described above (Fig. 1D). The promoters controlling the expression of the hygromycin and neomycin resistance genes are provided only upon recombination at the *FRT* and *loxP2272/loxP* sites, respectively. This ensures that each cell contains a single copy of the HA-tagged and Flag-tagged hERG1 genes (Fig. 1E), and allows the polyclonal selection of the double-stable cell line.

### Quantitative PCR

Quantitative real-time PCR was used to confirm the copy number of the *FRT* and *loxP2272/loxP* target sites within the Flp-Cre cell line. Genomic DNA from Flp-Cre clone #14 was isolated with the Gentra Puregene kit (Qiagen) following the manufacturer's protocol. PCR primers were specific to sequences within the *FRT* (zeocin resistance gene) and *loxP2272/loxP* (GFP) cassette. Primer sequences are: zeocin forward (5′-GTT GAC CAG TGC CGT TCC-3′), zeocin reverse (5′-TGA ACA GGG TCA CGT CGT C-3′), GFP forward (5′-GCG AGG GCG ATG CCA CCT AC-3′), and GFP reverse (5′-TCG GGG TAG CGG CTG AAG CA-3′). To take into account the amplification efficiency of each primer set, we used a plasmid DNA containing the cDNAs of zeocin and GFP as template for generating standard curves for each primer set. By using a plasmid in which the zeocin and GFP cDNA fragments are at 1∶1 ratio, equal quantities can be assigned to each dilution point of the standard curves [38]. PCR was performed on the MX300P real-time PCR machine (Stratagene, La Jolla, CA) using Power SYBR Green PCR Master Mix (Applied Biosystems, Foster City, CA). After denaturing at 95°C for 10 min, the reaction was run for 40 cycles with denaturation at 95°C for 30 s, annealing at 55°C for 30 s and primer extension at 72°C for 30 s.

### Electrophysiology

Membrane currents were recorded in whole cell configuration using suction pipettes, at ∼22°C, as previously described [11]. An Axopatch-200B amplifier was used to record membrane currents and the computer software pCLAMP8 was used to analyze current signals. Data are presented as mean ± SEM and analyzed by Student's *t*-test. *P*<0.05 is considered statistically significant.

### Western blot and immunoprecipitation

Western blot analysis and immunoprecipitation were performed as previously described [31], [39]. Briefly, proteins from whole cell lysates were subjected to SDS-PAGE, transferred onto nitrocellulose membranes, detected with the anti-HA and the anti-Flag antibody and visualized with the Plus-ECL (PerkinElmer, Waltham, MA) detection kit. The expression level of neomycin phosphotransferase II encoded by the neomycin resistant gene was used as an internal control for normalizing the relative expression of hERG isoform proteins. The neomycin resistance gene is a component of the pUC18/*loxP* expression vector and is stably integrated at the *loxP2272/loxP* site of Flp-Cre cells. The polyclonal anti-neomycin phosphotransferase II antibody (Milipore, Billerica, MA) was used at a 1∶300 dilution. In the immunoprecipitation experiments, cells were lysed with immunoprecipitation buffer (10 mM Tris-HCl, pH 8.0, 150 mM NaCl, 5 mM EDTA, 1% Triton X-100, 1 mg/ml BSA and protease inhibitors), hERG1 channels were immunoprecipitated with the anti-HA antibody and detected by western blot with the anti-Flag and the anti-HA antibody. hERG1 protein was quantified by densitometry with ImageJ software [40].

### Immunofluorescence microscopy

The characterization of wild-type and mutant hERG1 channel trafficking by immunofluorescence has been described previously [41]. Briefly, Flp-Cre cell lines stably expressing HA-tagged hERG1a and Flag-tagged hERG1a or Flag-tagged hERG1a_USO_ channels were fixed with 4% paraformaldehyde, blocked with PBS containing 5% goat serum and 0.2% Triton X-100, and incubated with monoclonal anti-HA and polyclonal anti-Flag antibody for one hour at room temperature. The cells were then washed and probed with Alexa Fluor 488-conjugated goat anti-mouse and Alexa Fluor 594-conjugated goat anti-rabbit secondary antibody (Molecular Probes, Eugene, OR). Images were acquired with a Zeiss Axioskop 2 microscope.

## Supporting Information

Figure S1 Flp-Cre cells co-expressing HA-tagged hERG1a (1a) and Flag-tagged wild-type (WT) or mutant (Mut) 1a or hERG1a_USO_ (1a_USO_) channels were lysed using an immunoprecipitation buffer containing the NP40 detergent. hERG1 channels were detected by western blot with the anti-HA and the anti-Flag antibody (upper two panels). Co-assembly of hERG1 isoforms was determined by immunoprecipitation with the anti-HA antibody and detection with the anti-Flag and the anti-HA antibody (lower three panels). Results shown are representative of three independent experiments.
(TIF)

Figure S2 Immunofluorescence staining of Flp-Cre cells stably expressing HA-tagged hERG1a and Flag-tagged hERG1a-G628S or Flag-tagged hERG1a_USO_-G628S channels. The phase contrast image, the monoclonal anti-HA staining, the polyclonal anti-Flag staining and the merged fluorescence signal from the anti-HA and anti-Flag staining are shown. Bar 20 µm, applies to all panels.
(TIF)
