# Supplementary figures and images for: Isoform-Specific Dominant-Negative Effects Associated with hERG1 G628S Mutation in Long QT Syndrome
Source: PLoS One. 2012 Aug 2;7(8):e42552. doi: 10.1371/journal.pone.0042552 (PMC3411645; doi:10.1371/journal.pone.0042552)

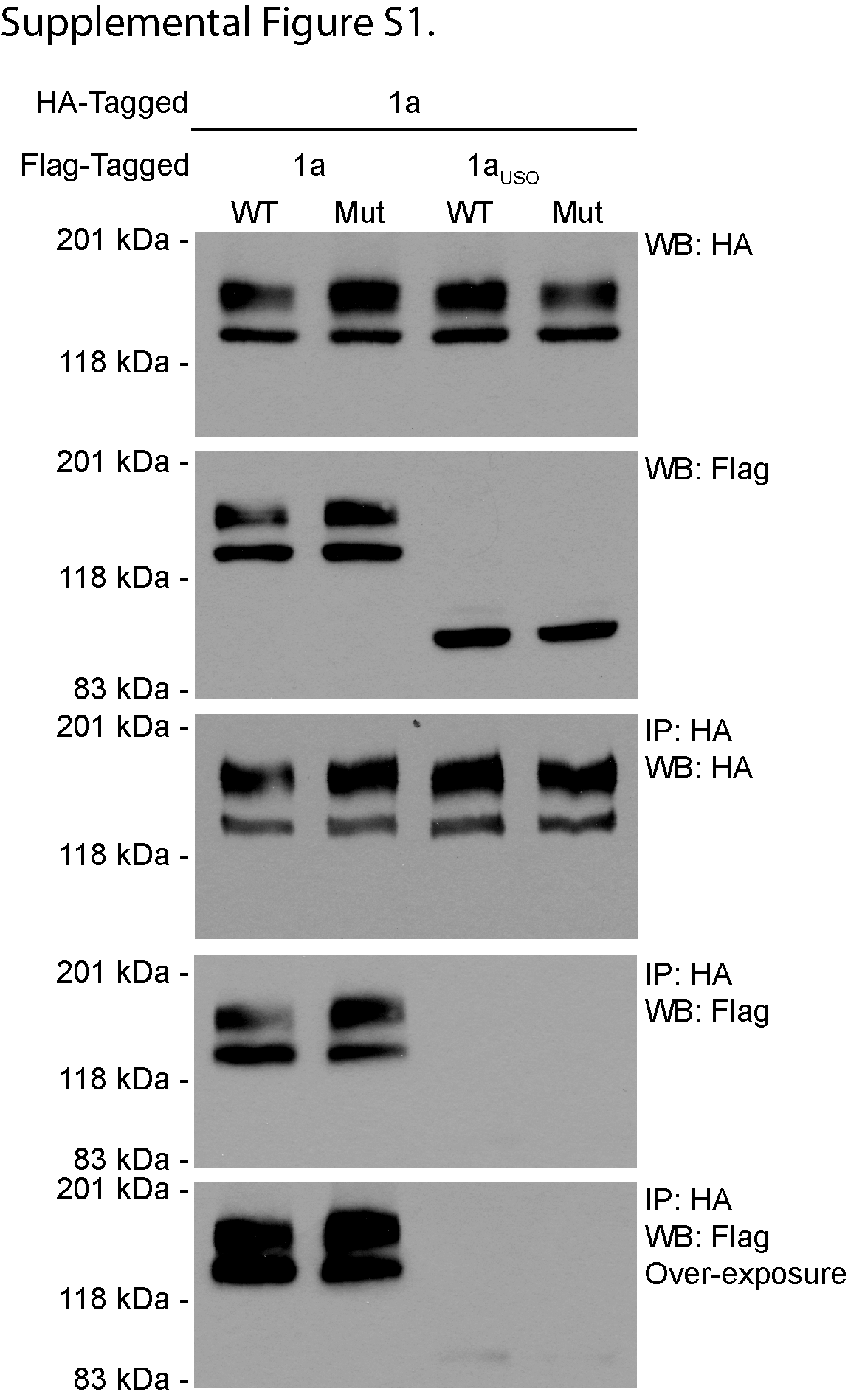

Supplement: Figure S1 — Flp-Cre cells co-expressing HA-tagged hERG1a (1a) and Flag-tagged wild-type (WT) or mutant (Mut) 1a or hERG1aUSO (1aUSO) channels were lysed using an immunoprecipitation buffer containing the NP40 detergent. hERG1 channels were detected by western blot with the anti-HA and the anti-Flag antibody (upper two panels). Co-assembly of hERG1 isoforms was determined by immunoprecipitation with the anti-HA antibody and detection with the anti-Flag and the anti-HA antibody (lower three panels). Results shown are representative of three independent experiments. (TIF) [file pone.0042552.s001.tif]

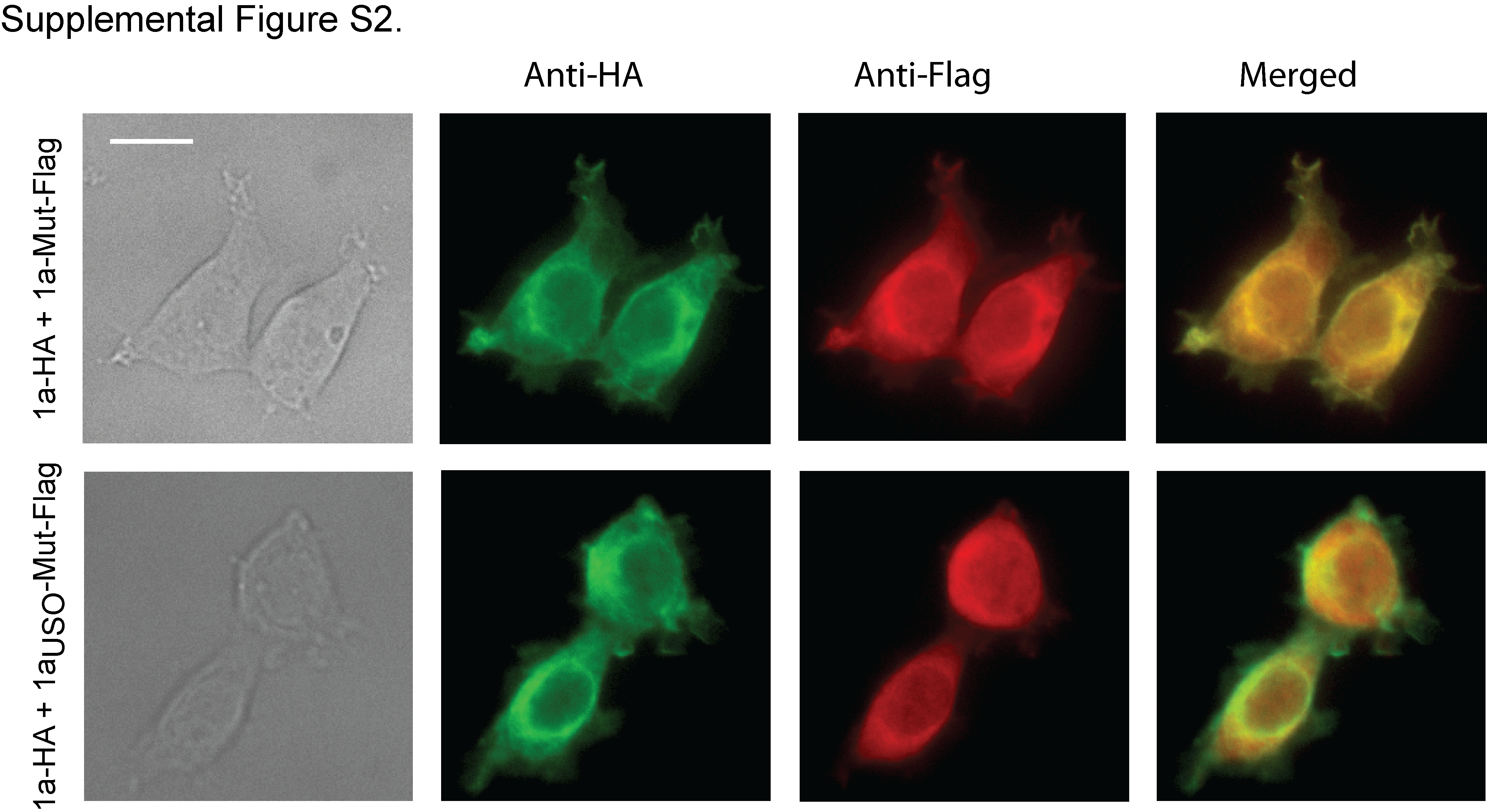

Supplement: Figure S2 — Immunofluorescence staining of Flp-Cre cells stably expressing HA-tagged hERG1a and Flag-tagged hERG1a-G628S or Flag-tagged hERG1aUSO-G628S channels. The phase contrast image, the monoclonal anti-HA staining, the polyclonal anti-Flag staining and the merged fluorescence signal from the anti-HA and anti-Flag staining are shown. Bar 20 µm, applies to all panels. (TIF) [file pone.0042552.s002.tif]
